# SOCIOCULTURAL VARIABILITY IN SELF-REPORTED COGNITIVE IMPAIRMENT AMONG OLDER LATINOS IN THE UNITED STATES

**DOI:** 10.1093/geroni/igz038.2167

**Published:** 2019-11-08

**Authors:** Marc A Garcia, David F Warner, Catherine Garcia

**Affiliations:** 1 University of Nebraska, Lincoln, Nebraska, United States; 2 University of Southern California, Los Angeles, California, United States

## Abstract

Cognitive impairment is a major public health concern in the United States. Research indicates cognitive impairment is higher for older U.S. Latinos than non-Latino whites, due in part to Latinos having longer life expectancy, lower educational attainment, and a higher prevalence of diabetes and cardiovascular disease. Prior studies on cognition have largely examined “Latinos” as a monolithic group. However, Latinos are heterogeneous in composition with unique socio-cultural characteristics based on nativity and country of origin. Accordingly, we used data from the 1997-2017 National Health Interview Survey (NHIS) to document age-specific trends in in self-reported cognitive impairment among US-born Mexican, foreign-born Mexican, island-born Puerto Rican, foreign-born Cuban, and non-Latino white adults aged 60 and older. Given the repeated cross-sectional nature of these data, we estimated hierarchical age period–cohort (HAPC) cross-classified random-effects model (CCREM) to isolate age trends in self-reported cognitive impairment across Latino subgroups and non-Latino whites. Results indicate significant heterogeneity among Latino subgroups, with island-born Puerto Ricans exhibiting the highest rates of cognitive impairment and foreign-born Cubans the lowest. Conversely, US-born and foreign-born Mexicans exhibited rates in between these two. All Latino subgroups statistically differed from non-Latino whites. Socio-demographic controls account for approximately 33%-45% of the disparity, but fully account for foreign-born Cubans and non-Latino whites differences. These findings indicate the importance of considering nativity and country of origin when assessing cognitive outcomes among older Latinos. Understanding minority and immigrant differences in cognitive impairment has implications for the development and implementation of culture-appropriate programs to promote healthy brain aging.

